# Effect of high‐frequency membrane potential alternation between depolarization and hyperpolarization on dorsal root ganglion neurons of rats

**DOI:** 10.14814/phy2.15582

**Published:** 2023-01-25

**Authors:** Zhijun Shen, Jonathan Beckel, William C. de Groat, Changfeng Tai

**Affiliations:** ^1^ Department of Urology University of Pittsburgh Pittsburgh Pennsylvania USA; ^2^ Department of Pharmacology and Chemical Biology University of Pittsburgh Pittsburgh Pennsylvania USA; ^3^ Department of Bioengineering University of Pittsburgh Pittsburgh Pennsylvania USA

**Keywords:** block, high frequency, nerve, patch clamp, rat

## Abstract

The purpose of this study was to determine how sensory neurons respond to high‐frequency membrane potential alternation between depolarization and hyperpolarization. Membrane currents were recorded from dissociated dorsal root ganglion (DRG) neurons of adult rats using the whole cell patch clamp technique in voltage clamp mode. Stepwise depolarization of the membrane was applied first to determine the threshold membrane potential for inducing an action potential (AP) current. Then, membrane potential alternation between depolarization (to +20 mV) and hyperpolarization (to −110 mV) was applied to the neuron for 10 s at different frequencies (10 Hz to 1 kHz). The tested DRG neurons had APs of either a long duration (>10 ms) or a short duration (<10 ms). Membrane potential alternation at ≥500 Hz completely disrupted the AP generation, disabled the ion channel gating function, and produced membrane current alternating symmetrically across zero. Replacing extracellular sodium with potassium increased the amplitude of the membrane current response and caused the membrane current to be larger during hyperpolarization than during depolarization. These results support the hypothesis that high‐frequency biphasic stimulation blocks axonal conduction by driving the potassium channel open constantly. Understanding neural membrane response to high‐frequency membrane potential alternation is important to reveal the possible mechanisms underlying axonal conduction block induced by high‐frequency biphasic stimulation.

## Graphical abstract

Our study using dissociated DRG neurons of rats revealed that high frequency (≥500 Hz) membrane potential alternation between depolarization and hyperpolarization could disrupt action potential generation, disable ion channel gating function, and produce a symmetric membrane current waveform. The high frequency membrane potential alternation produced sodium channel inactivation while driving the potassium channel open constantly. These results provide experimental data supporting the hypothesis that high frequency biphasic stimulation above 4 kHz can produce axonal conduction block by driving the potassium channel to open constantly, thereby eliminating the delay between sodium and potassium currents, preventing AP generation, and causing conduction block.

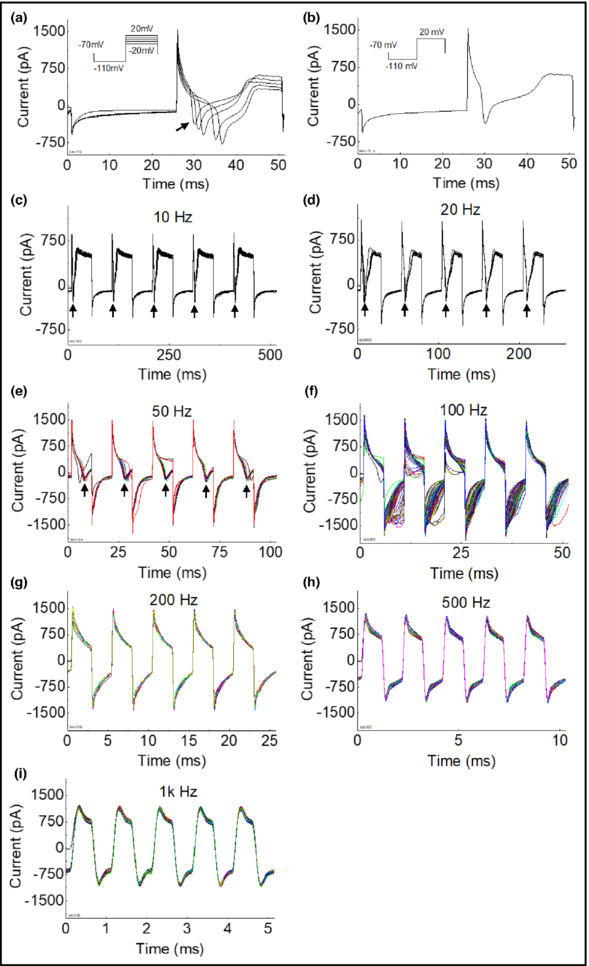

## 1 INTRODUCTION

Nerve conduction block by high‐frequency (kHz) biphasic stimulation (HFBS) has been known for 80 years (Reboul & Rosenblueth, 1939; Rosenblueth & Reboul, 1939). Recently, this nerve block method has been used clinically to treat obesity by blocking the vagus nerve (Apovian et al., 2017) or to treat post‐amputation limb pain by blocking the sciatic nerve (Soin et al., 2015). HFBS was also investigated in animal studies to block the pudendal nerve for potential treatment of urinary dysfunction after spinal cord injury (Chen et al., 2021). Despite the long history of HFBS‐induced nerve conduction block and its recent success in clinical application, the mechanism of action is still unknown. Understanding the mechanism is important to improve the kHz nerve block therapy or develop new electrical nerve block methods.

Previous animal studies (Bowman & McNeal, 1986; Reboul & Rosenblueth, 1939; Rosenblueth & Reboul, 1939) have shown that a minimal stimulation frequency of 4 kHz is required for HFBS to induce a reliable nerve conduction block. Our modeling analyses (Tai et al., 2005; Zhang et al., 2006) further reveal that the requirement for a minimal frequency is determined by the axonal ion channel kinetics. During HFBS, the axonal membrane is alternatively depolarized and hyperpolarized. When the frequency of this alternation is above 4 kHz, HFBS can drive the potassium channel open constantly, eliminating the delay between sodium and potassium currents when the action potential (AP) arrives, and thereby resulting in a conduction block (Tai et al., 2005; Zhang et al., 2006). To test this hypothesis and confirm our computer modeling results, experimental studies are needed to understand how the neural membrane responds to high‐frequency membrane potential alternation between depolarization and hyperpolarization.

In this study, the neural membrane response to high‐frequency alternation between depolarization and hyperpolarization was investigated in isolated dorsal root ganglion (DRG) neurons of rats using the whole cell patch clamp technique. DRG neurons were used instead of axons due to the difficulty in recording from the axonal node with the patch clamp technique. Although the ion channel kinetics of soma and axonal membranes are different, this initial study of the response of the DRG soma to high‐frequency biphasic electrical stimulation could still provide valuable information about the influence of HFBS on ion channels and insight into the possible mechanisms underlying HFBS nerve block.

## 2 MATERIALS AND METHODS

### 2.1 DRG preparation

DRG neurons were obtained from young adult (2 months old) Sprague Dawley male rats (*N* = 9) euthanized in a CO_2_ chamber. A segment of the spinal column from T7 to L6 was surgically removed and placed in a dissection dish on ice containing DMEM (Sigma–Aldrich). DRGs were harvested under a dissection microscope and transferred to culture media on ice in a 30‐mm culture dish. After removing excessive connective tissue from the DRGs, they were minced with a pair of fine eye surgical scissors. The DRG tissues were transferred to another dish with enzymatic digestive solution and stored for 30 min in an incubator (95% O_2_, 5% CO_2_, 37°C) for dissociation. The DRG tissues were then subjected to a cleaning process with culture media 3 times, seeded on 5 × 5 mm glass cover slips coated with poly‐l‐lysine, immersed in culture media in a 30‐mm dish, and kept in the incubator for 2‐h before the patch clamp experiment.

### 2.2 Patch clamp recording setup

The patch clamp system consists of an Axon 700A amplifier, an Axon Digidata 1320a data acquisition system, and a Window‐based personal computer with pClamp 9 software (Molecular Devices, San Jose, CA, USA). A custom‐made plastic recording chamber was mounted on the stage of an Olympus inverted microscope with a gravity‐fed perfusion system. A Burleigh micromanipulator was equipped for handling a pipette holder connected to the recording head stage. Borosilicate glass micopipettes with resistance around 2 MΩ when filled with a pipette solution containing 140 mM KCl were produced from glass capillaries (1B150F‐4, WPI, Lawrence, PA, USA) by a Sutter‐97 puller (SU‐P97, Sutter Instrument, Novato, CA, USA). DRG neurons on a glass cover slip were placed in the recording chamber and constantly perfused with a physiological solution during the experiment. Whole‐cell voltage clamp recording configuration was established after making a giga seal between the pipette microelectrode and neuron membrane. Membrane signals were sampled at 50 kHz and saved to the disc on a personal computer for offline analysis using Origin software (Origin Lab, Northampton, MA, USA).

### 2.3 Solution composition

Physiological solution: 138 mM NaCl, 5 mM KCl, 3 mM CaCl_2_, 1 mM MgCl_2_, 10 mM HEPES, 0.3 mM KH_2_PO_4_, 0.3 mM NaH_2_PO_4_, 4 mM NaHCO_3_, 6 mM glucose, pH 7.2 with 1 N NaOH. Pipette solution: 140 mM KCl, 5 mM NaCl, 1 mM CaCO_2_, 2 mM MgCl_2_, 10 mM HEPES, 1 mM EGTA, 2 mM Mg‐ATP, pH 7.2 with 1 N KOH. High K^+^‐based solution: 140 mM KCl, 3 mM CaCl_2_, 1 mM MgCl_2_, 10 mM HEPES, 0.3 mM KH_2_PO_4_, 0.3 mM KH_2_PO_4_, 4 mM NaHCO_3_, 6 mM glucose, pH 7.2 with 1 N KOH. Culture media: Neurobasal A Media (Invitrogen), 5% B27 (Invitrogen), and 1% PSN (Sigma–Aldrich). Digestive solution: 1 mg/mL Trypsin (Worthington Biochemical) and 2 mg/ml Collagenase type 4 (Worthington Biochemical) were added to the culture media. All Chemicals were obtained from Sigma–Aldrich.

### 2.4 Experimental protocol

**FIGURE 1:**
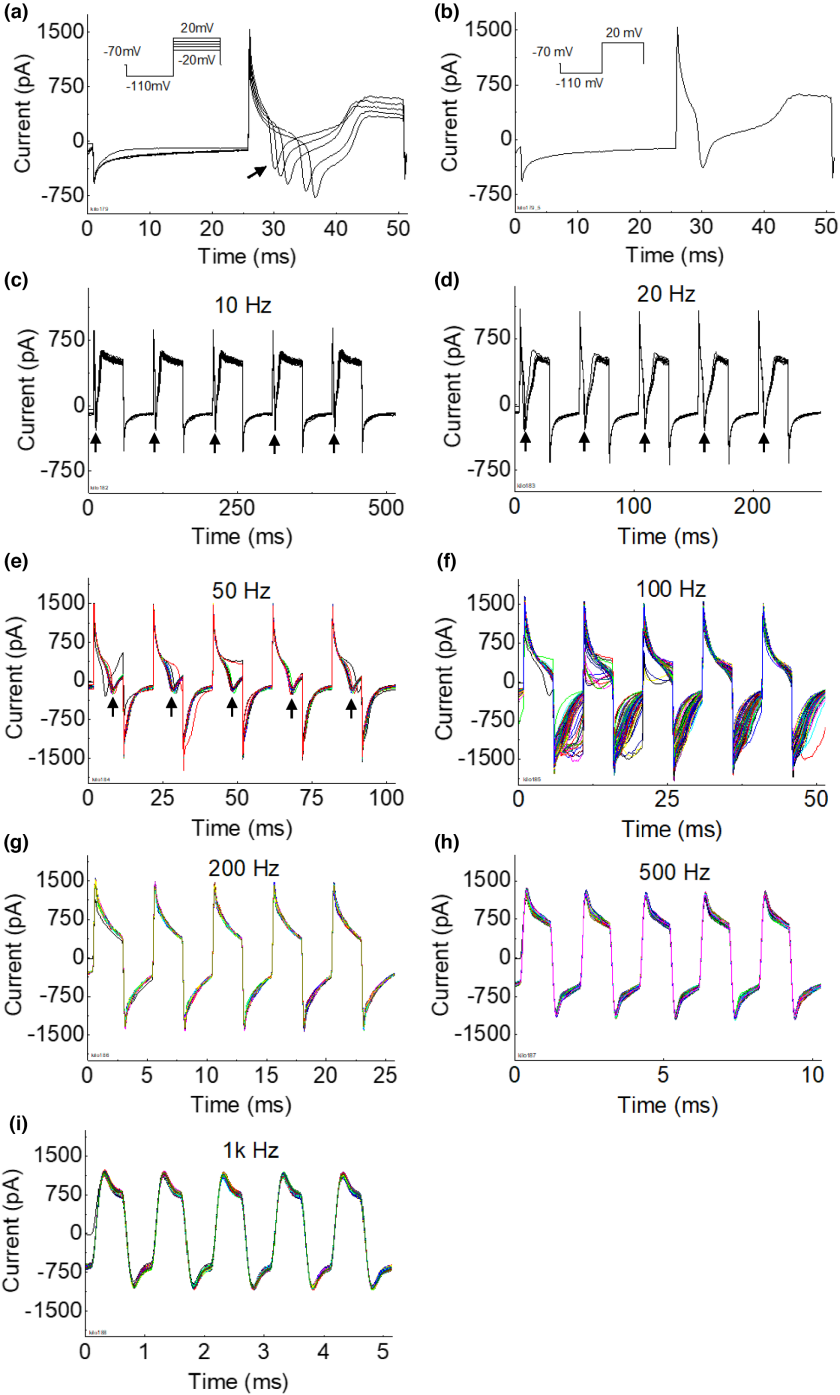
Membrane current responses to membrane potential alternation in a neuron with a long duration (>10 ms) action potential (AP) current. (a) The AP currents elicited by stepwise depolarization (see the insert for stimulation protocol). (b) A single AP current selected from figure a (indicted by the arrow in a) that was elicited by a pre‐hyperpolarization (−110 mV) and then stepping to a depolarization level of +20 mV. (c–i) Membrane current responses to different frequencies (10 Hz to 1 kHz) of membrane potential alternation (10 s) between depolarization (to +20 mV) and hyperpolarization (to −110 mV). The arrows indicate the peaks of AP currents. Each figure (c–i) showed 10‐s membrane current response that was divided into multiple segments and then overlayed together for plotting. The duration of each response segment was determined by the alternation frequency to cover five periods of the alternation.

**FIGURE 2:**
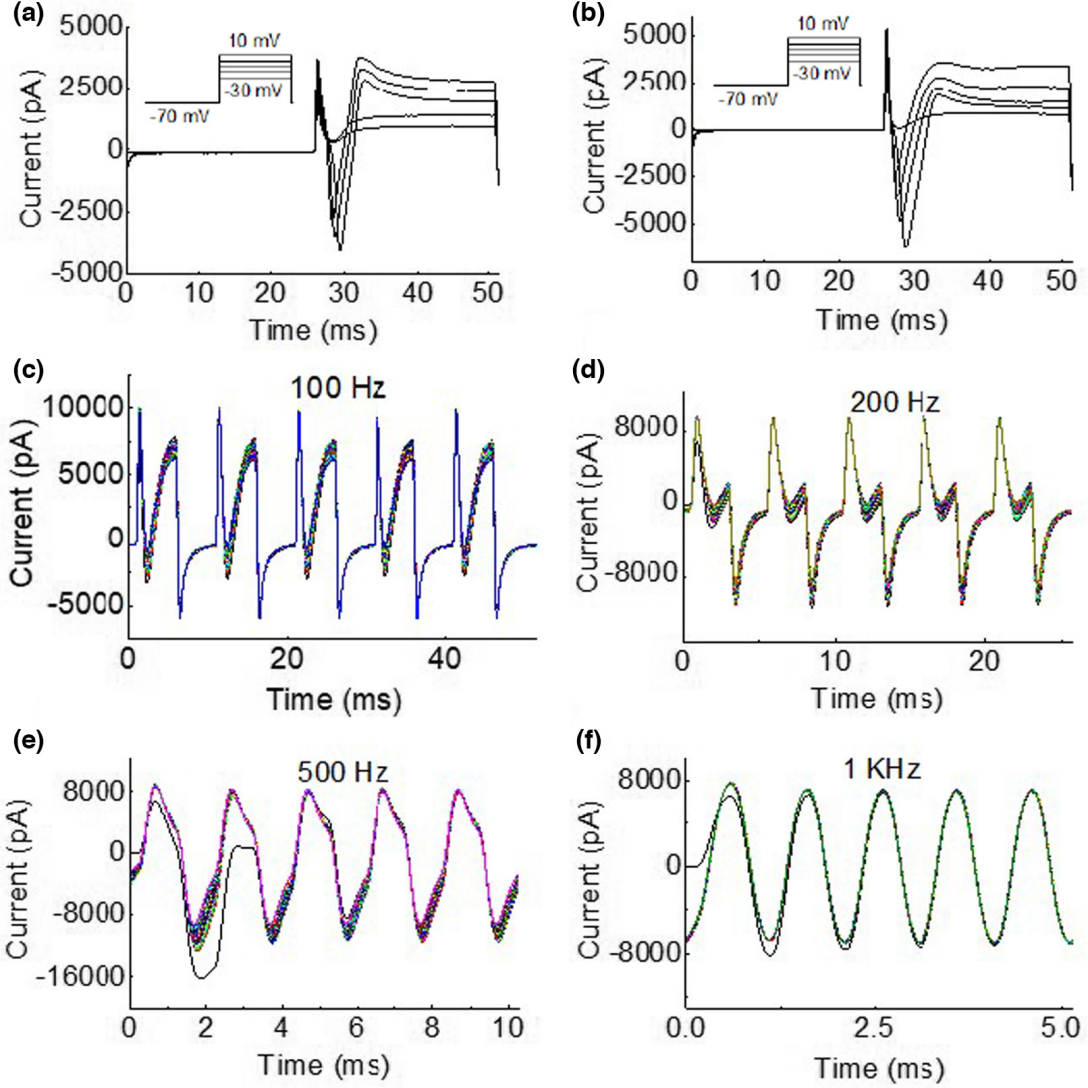
Membrane current responses to membrane potential alternation in a neuron with a short duration (<10 ms) action potential (AP). (a) The AP currents elicited by stepwise depolarization (see the insert for stimulation protocol). (b) The AP currents elicited after the different frequency membrane potential alternation. (c–f) Membrane current responses to different frequencies (100 Hz to 1 kHz) of membrane potential alternation (10 s) between depolarization (to +20 mV) and hyperpolarization (to −110 mV). Each figure (c–f) shows a 10‐s membrane current response that was divided into multiple segments and then overlayed together for plotting. The duration of each response segment was determined by the alternation frequency to cover five periods of the alternation.

The DRG neuron membrane potential was held at −70 mV during the experiment when no test was being performed. At the beginning of the experiment, AP currents were evoked by a stepwise depolarization. From the initial holding potential of −70 mV, the membrane was first hyperpolarized to −110 mV and then stepped to a depolarization level up to +50 mV in 10 mV increments (Figure 1a), or directly stepped to a depolarization level from the −70 mV (Figure 2a). After observation of the AP currents, a range of membrane potentials was chosen to alternatively depolarize and hyperpolarize the membrane between +20 mV and −110 mV. The depolarization potential (+20 mV) and the hyperpolarization potential (−110 mV) were chosen based on our previous modeling studies (Tai et al., 2005; Zhang et al., 2006) showing that HFBS could produce an axonal conduction block by alternating the membrane potential between a hyperpolarization 40 mV above the resting potential and a depolarization 90 mV below the resting potential. The alternation between depolarization and hyperpolarization was applied to the neuron membrane (*N* = 18 neurons) at a certain frequency (10 Hz to 1 kHz) for 10 s while the membrane current response was recorded. The membrane potential was held at −70 mV for a 1‐s period between the different frequency membrane alternations. In 11 of the 18 neurons, the physiological solution (138 mM NaCl, 5 mM KCl) in the bath was replaced by high K^+^‐based solution (140 mM KCl, 0 mM NaCl) that had the same K^+^ concentration as the pipette solution (140 mM KCl, 5 mM NaCl). The 10‐s alternation between depolarization and hyperpolarization was applied again at different frequencies to determine the membrane current response in the high K^+^‐based solution. All experiments were performed at room temperature (about 20–25°C).

### 2.5 Data analysis

The 10‐s membrane current response was divided into multiple segments based on the alternation frequency so that each segment included five periods of membrane potential alternations. These segmented current responses were then overlayed for plotting at each alternation frequency to determine the effect of frequency. To show the similar time course of the membrane current responses in different neurons, the membrane current was normalized to the peak current in each test for each neuron and then plotted together for comparison.

## 3 RESULTS

### 3.1 Membrane current response to membrane potential alternation

The AP currents recorded in this study had either a long duration (>10 ms, Figure 1a, *N* = 5) or a short duration (<10 ms, Figure 2a, *N* = 13). For the neuron with a long‐duration AP (Figure 1b), the AP current was clearly visible during the depolarization when the frequency of membrane potential alternation was below 20 Hz (see the arrows in Figure 1c,d). At 50–100 Hz, the AP current was disrupted by hyperpolarization (Figure 1e) and the membrane current during hyperpolarization became variable during the 10‐s period of membrane potential alternation (Figure 1f). As the frequency increased to ≥200 Hz (Figure 1g–i), the neuron did not generate an observable AP current during depolarization and the hyperpolarization current became stable during the 10‐s alternation. It is worth noting that as the frequency of membrane potential alternation increased the membrane current response changed gradually from an asymmetric waveform between depolarization and hyperpolarization (Figure 1c–f) to a symmetric waveform (Figure 1g–i).

For the neuron with a short‐duration AP current (Figure 2a), the disruption of AP current occurred at a higher frequency of 200 Hz (Figure 2d) when compared to the neuron with a long‐duration AP current (Figure 1e,f). However, as the frequency of membrane potential alternation further increased (500 Hz to 1 kHz, Figure 2e,f), the membrane current response also became a symmetric waveform alternating between depolarization and hyperpolarization. It is worth noting that a negative shift of the membrane current caused by the initial AP was still visible during the first 5 ms of the 10‐s membrane potential alternation at 500 Hz (Figure 2e). About 10 s after applying the different frequency membrane potential alternations, stepwise depolarizations induced AP currents with a slightly reduced threshold (Figure 2b).

**FIGURE 3:**
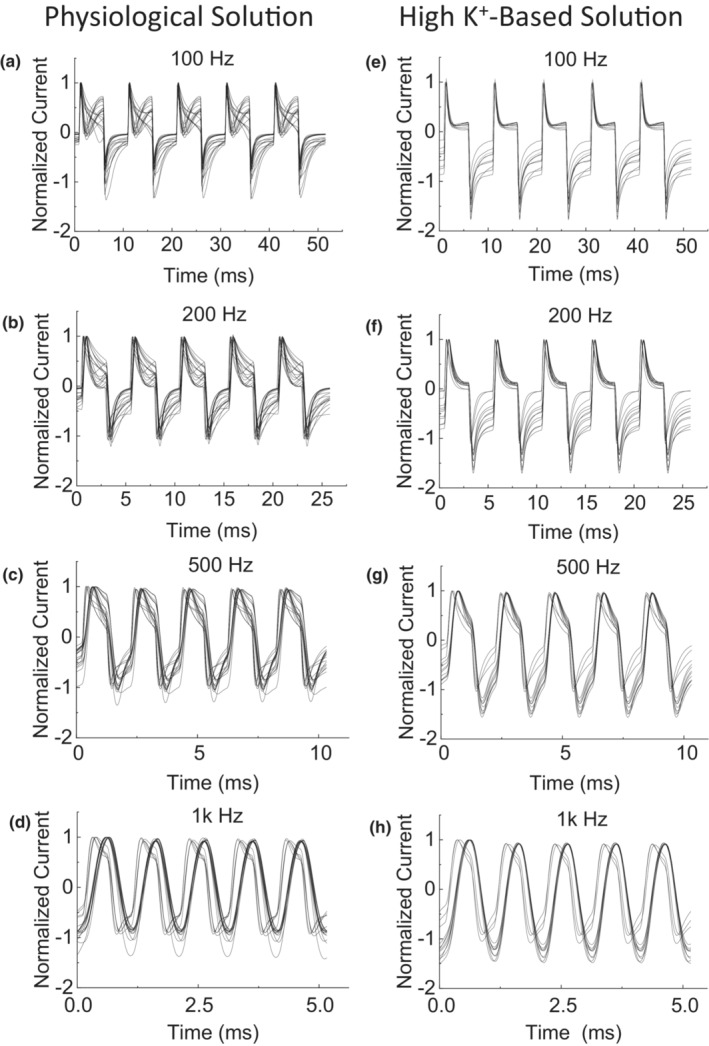
Normalized membrane current responses were plotted for physiological solution (a–d, *N* = 18 neurons) or high K^+^‐based solution (e–h, *N* = 11 neurons). For each neuron, only the last five periods of alternation of the 10‐s membrane current response were plotted.

Figure 3a–d shows the normalized membrane current responses from 18 neurons in physiological solution. For neurons with either short (*N* = 13) or long (*N* = 5) duration AP current, the membrane current responses became more symmetric at the alternation frequency above 500 Hz (Figure 3a,c,d).

### 3.2 Effect of replacing extracellular sodium with potassium

**FIGURE 4:**
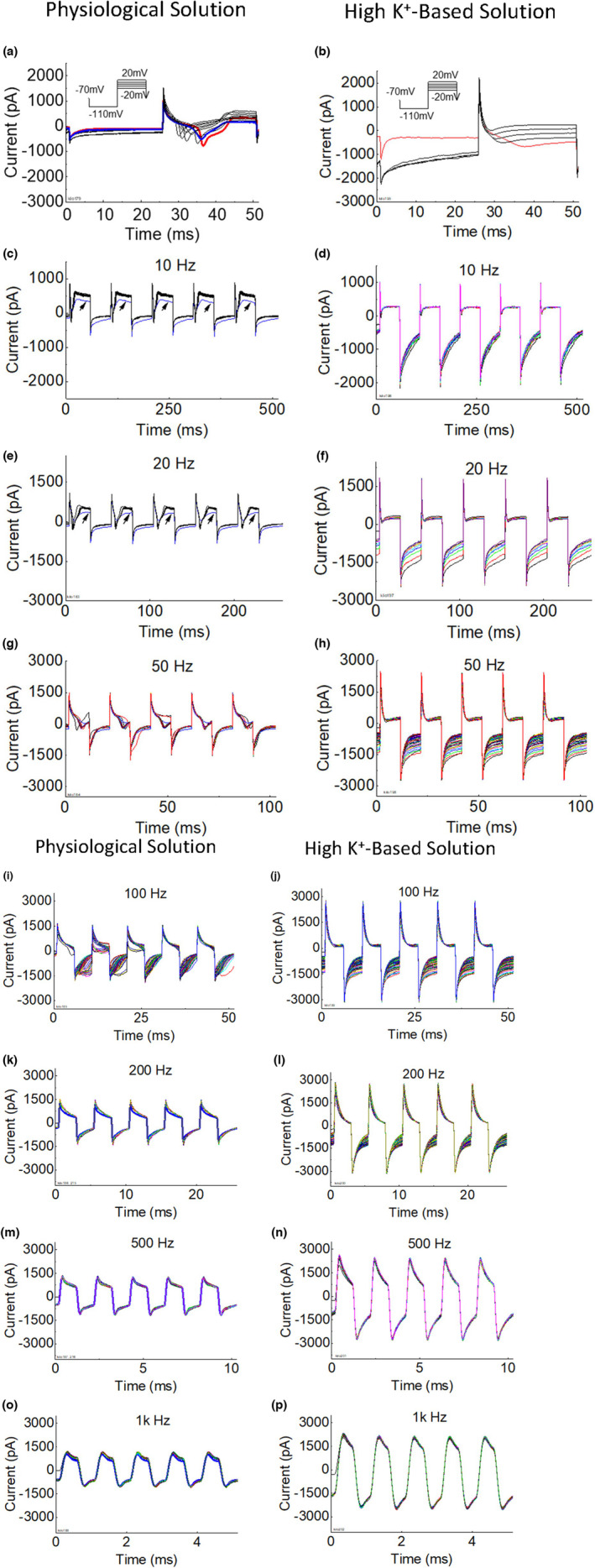
Effect of replacing extracellular Na^+^ with K^+^ on membrane current response to membrane potential alternation at different frequencies. The responses in the left column were obtained with the neuron in physiological solution (138 mM NaCl, 5 mM KCl). The responses in the right column were obtained from the same neuron after replacing the physiological solution with the high K^+^‐based solution (140 mM KCl, 0 mM NaCl). The same membrane potential alternation protocol (depolarization to +20 mV and hyperpolarization to −110 mV) was used with both solutions. The left column also includes the responses after changing the high K^+^‐based solution back to the physiological solution, which results in a smaller action potential current (blue vs. red in a) and minor changes in responses to the membrane potential alternation (only observable in c and e as indicated by the arrows). *Note*: These responses are from the same neuron shown in Figure 1.

Figure 4 shows responses of the same neuron to membrane potential alternation with physiological solution in the recording bath (left column in Figure 4) and after the bath solution was changed to the high K^+^‐based solution (right column in Figure 4). The AP was eliminated by replacing the extracellular Na^+^ with K^+^ (Figure 4a,b). In addition, the hyperpolarization current was much larger in the high K^+^‐based solution than in the physiological solution after the first stepwise depolarization (Figure 4a,b). As the frequency increased above 500 Hz, the membrane current response became more symmetric and very repeatable during each alternation period for the entire 10 s of membrane potential alternation for both extracellular solution conditions (Figure 4m–p). It is worth noting that the maximal current was larger during both depolarization and hyperpolarization in the high K^+^‐based solution than in the physiological solution (Figure 4m–p). After changing the high K^+^‐based solution back to the physiological solution, the AP current re‐appeared with a smaller amplitude (blue vs. red, Figure 4a). The neuron responses to different frequency membrane potential alternations were very similar before and after high K^+^‐based solution treatment with only minor differences observable at the low frequencies 10–20 Hz (as indicated by the arrows in Figure 4c,e).

Figure 3e–h show the normalized membrane current responses from 11 neurons in high K^+^‐based solution. At the frequencies ≥500 Hz, most of the neurons (*N* = 8) still showed an un‐symmetric response with a larger membrane current during hyperpolarization than during depolarization (Figure 3g,h).

## 4 DISCUSSION

This study using dissociated DRG neurons of rats revealed that high‐frequency (≥500 Hz) membrane potential alternation between depolarization (+20 mV) and hyperpolarization (−110 mV) can produce a membrane current alternating symmetrically across zero (Figures 1, 2 and 3a–d). At a frequency between 50 and 200 Hz, the membrane potential alternation started to disrupt the AP current generation (Figures 1 and 2). Replacing the extracellular sodium with potassium increased the amplitude of the membrane current (Figure 4) that was alternating un‐symmetrically across zero with the current during hyperpolarization larger than during depolarization (Figure 3e–h). These results indicate that potassium channels might play an important role in the membrane current response to high‐frequency (≥500 Hz) membrane potential alternation.

Our previous modeling analyses (Tai et al., 2005; Zhang et al., 2006) have shown that the minimal frequency of 4 kHz for HFBS to induce axonal conduction block in animal studies (Bowman & McNeal, 1986; Reboul & Rosenblueth, 1939; Rosenblueth & Reboul, 1939) is determined by potassium channel kinetics. As the HFBS frequency increases to 4 kHz, the axonal membrane potential alternation between depolarization and hyperpolarization is fast enough to drive the potassium channels to open constantly, which eliminates the delay between sodium and potassium current generation when the AP arrives, thereby preventing the AP initiation (Tai et al., 2005; Zhang et al., 2006). Since DRG neurons were used in this study instead of axons due to the technique difficulty in patch clamping an axon, the difference in AP durations between neuron and axon should be considered. Since the AP duration of an axon is about 1 ms but is about 10 ms for a neuron (Figures 1a and 2a), we estimate that the ion channels open and close about 10 times slower in neurons. This estimation predicts that a minimal frequency of about 400 Hz might be fast enough to disrupt the membrane gating mechanism in these neurons. This estimation agrees well with our current study showing that a minimal frequency of 200–500 Hz is needed to disrupt the neuron AP current and produce a symmetric membrane current waveform (Figures 1 and 2). The membrane current waveform changes from asymmetric to symmetric as the frequency increases to ≥500 Hz (Figure 3a–d), indicating that the ion channel gating mechanism cannot follow the fast frequency and thus gradually loses gating function. Therefore, the sodium and potassium channels must be either closed or open constantly at the fast frequencies ≥500 Hz. Since the amplitude of the alternating membrane current is sensitive to the change in extracellular potassium concentration (Figure 4), it is highly likely that the high‐frequency alternation of membrane potential produces sodium channel inactivation while driving the potassium channel open constantly. The initial activation of sodium channels can be observed in Figure 2e where at 500 Hz the membrane current exhibits an initial negative shift during the first 5 ms of the 10‐s membrane alternation. This initial negative shift indicates that the sodium channel was activated initially to allow an inward sodium current but was then inactivated quickly by the high‐frequency alternation to produce a symmetric membrane current waveform without ion channel gating function. Furthermore, with a high extracellular potassium concentration the inward membrane current during hyperpolarization was larger than the outward membrane current during depolarization even after the ion channel gating function was lost at the fast frequencies ≥500 Hz (Figure 3e–h). This further indicates that the potassium channel was probably open constantly at this high frequency, allowing more potassium ions to flow inward than outward due to the high extracellular potassium concentration. Additional study is warranted to further investigate the effect of high‐frequency membrane potential alternation on sodium and potassium channel gating properties. Nevertheless, the results of our current study support the idea that high‐frequency alternation of membrane potential can drive potassium channels to open constantly to prevent AP generation.

It is possible that other ion currents such as calcium and chloride may also contribute to the symmetric membrane current in addition to sodium and potassium currents. Furthermore, the membrane capacitive current is certainly contributing to the symmetric membrane current by alternatively charging and discharging the membrane capacitor. More studies are needed to further investigate the composition of the symmetric membrane current to better understand the neural membrane response to high‐frequency alternation of the membrane potential.

This study investigated the responses to high‐frequency electrical stimulation by applying high‐frequency potential alternation directly across the neuronal membrane using the voltage clamp technique. Although this method is useful to study membrane current and ion channel gating properties in response to high‐frequency membrane potential alternation, HFBS in clinical applications to block nerve conduction is always applied extracellularly but not directly across the neural membrane (Apovian et al., 2017; Chen et al., 2021; Soin et al., 2015). Since kHz stimulation is also successful when applied to the spinal cord to treat chronic back pain (Al‐Kaisy et al., 2018; Hagedorn et al., 2020), investigation of neuronal response to extracellular application of HFBS using the patch clamp technique will certainly provide information to better understand the possible mechanisms of HFBS in the central nervous system where effects on neuronal perikarya and synaptic transmission as well as effects on axons are likely to be important.

In summary, this study using dissociated DRG neurons of rats revealed that high‐frequency (≥500 Hz) membrane potential alternation between depolarization and hyperpolarization could disrupt AP current generation, disable ion channel gating function, and produce a symmetric membrane current waveform (Figures 1, 2, 3). The high‐frequency membrane potential alternation probably produced sodium channel inactivation while driving the potassium channel open constantly. These results provide experimental data supporting the hypothesis that HFBS above 4 kHz can produce axonal conduction block by driving the potassium channel to open constantly, thereby eliminating the delay between sodium and potassium currents, preventing AP generation, and causing conduction block.

## FUNDING INFORMATION

This study is funded by the National Institute of Neurological Disorders and Stroke under grant R01NS109198.

## CONFLICT OF INTEREST

The authors have no conflict of interest.

## ETHICS STATEMENT

All experimental protocols and animal use in this study were approved by the Animal Care and Use Committee at the University of Pittsburgh.
